# Can Programming Frameworks Bring Smartphones into the Mainstream of Psychological Science?

**DOI:** 10.3389/fpsyg.2016.01252

**Published:** 2016-08-23

**Authors:** Lukasz Piwek, David A. Ellis

**Affiliations:** ^1^Division of Information, Decisions and Operations, School of Management, University of BathBath, UK; ^2^Department of Psychology, Lancaster UniversityLancaster, UK

**Keywords:** smartphones, mobile apps, digital sensors, behavioral informatics, mobile computing

## Abstract

Smartphones continue to provide huge potential for psychological science and the advent of novel research frameworks brings new opportunities for researchers who have previously struggled to develop smartphone applications. However, despite this renewed promise, smartphones have failed to become a standard item within psychological research. Here we consider the key issues that continue to limit smartphone adoption within psychological science and how these barriers might be diminishing in light of ResearchKit and other recent methodological developments. We conclude that while these programming frameworks are certainly a step in the right direction it remains challenging to create usable research-orientated applications with current frameworks. Smartphones may only become an asset for psychology and social science as a whole when development software that is both easy to use and secure becomes freely available.

Several recent papers have argued convincingly that smartphones will soon become a standard research tool amongst psychologists (Gan and Goh, 2016). For example, Miller (2012) suggested that smartphones would revolutionize psychology and behavioral science, concluding that the question is not whether psychology will make use of smartphones, but rather who, where, and when. By 2012, other disciplines had already been using smartphones extensively for many years in order to measure behavioral and cognitive processes. Computer scientists, for example, are using smartphone data for a diverse range of projects, although their focus is predominantly aimed at using machine learning to predict future behaviors and actions (Song et al., 2010; de Montjoye et al., 2013; Do and Gatica-Perez, 2014). Others are attempting to make smartphones cognitive'; by developing applications that can infer users' emotions (Lee and Park, 2012) or predict when users are talking about politics (even though content of communications is never known; Wei, 2014). Those within medicine are developing a range of psychological interventions' to support patients with mental health problems (Puiatti et al., 2011; Grünerbl et al., 2012; Donker et al., 2013; Gravenhorst et al., 2014; Ly et al., 2015), assist with behavior change to increase physical activity (Bort-Roig et al., 2014; Glynn et al., 2014) and facilitate weight loss (Allen et al., 2013; Carter et al., 2013). Another project, developed by Geographers, determined which locations in the UK were “happiest,” as well as the times, days, and situations when people were most happy (MacKerron and Mourato, 2013). While the advantages of using smartphones within research continue to be well documented, psychology and psychologists have often remained largely absent from the landscape. Exceptions include psychologists who are using text messaging or commercial systems to collect survey based data (e.g., Conner and Silvia 2015). However, from 11 examples of behavioral data collected via smartphones in Miller's manifesto, only two were published by psychologists (Killingsworth and Gilbert, 2010; Dufau et al., 2011). In both papers, smartphone apps were developed and the results demonstrated that smartphones provide an efficient method of collecting data. The researchers were able to make clear conclusions after reaching a wider demographic of participants, providing greater ecological validity. However, the majority of smartphone research from psychology labs currently tends to focus on self-report data about participants own smartphone use (e.g., Derks et al. 2014), rather than using the smartphone itself as a research tool (e.g., Andrews et al. 2015).

A typical approach when utilizing smartphones for research purposes is to develop a mobile application (or app) that can be downloaded by any participant from a commercial digital store or directly from research servers (Figures 1A,B). This app can than be used to deploy surveys, run experiments, and collect data from a rich selection of on-board smartphone sensors or other connected wearables (Figures 1C,D). Table 1 shows a number of frameworks and solutions that have been developed to facilitate the process of creating apps for research purposes, with the most common applications being ecological momentary assessment (Runyan and Steinke, 2015), surveying (Conner and Silvia, 2015), and data-logging (Ferreira et al., 2016). Most solutions presented in Table 1 can also be used to generate and deploy surveys, send notifications, collect data from devices' sensors or trigger “context-related” data collection depending on researchers' goal. Amongst recent additions to those solutions is ResearchKit (http://researchkit.org; Apple 2016b) which remains the only framework to have been developed by a major smartphone manufacturer. This alone could help prevent a second replication crisis within psychology by standardizing and validating data collection methods via the sharing of universal programming code, and unifying extensive distribution channels for smartphone-based studies. But what makes ResearchKit particularly unique for psychological research is the ability to create complex “active tasks,” which goes beyond surveying and data logging. This could, in turn, be used to create sophisticated and complex studies with smartphones. Examples of this already exist within medicine; for instance, the *mPower* app (http://parkinsonmpower.org) uses the iPhone's sensors to measure and track Parkinsons' patients' symptoms, including tremor, balance and gait, certain vocal characteristics, and memory. Researchers behind mPower implemented not only a surveying and sensor-logging paradigm, but also a range of experimental tasks where classic clinical tests for Parkinson were innovatively adapted for a smartphone interface. Also, thanks to popularity and exposure of App Store (where the ResearchKit apps are deployed) mPower sparked the largest single study on Parkinson within only few days following its release (Apple, 2016a).

**Figure 1:**
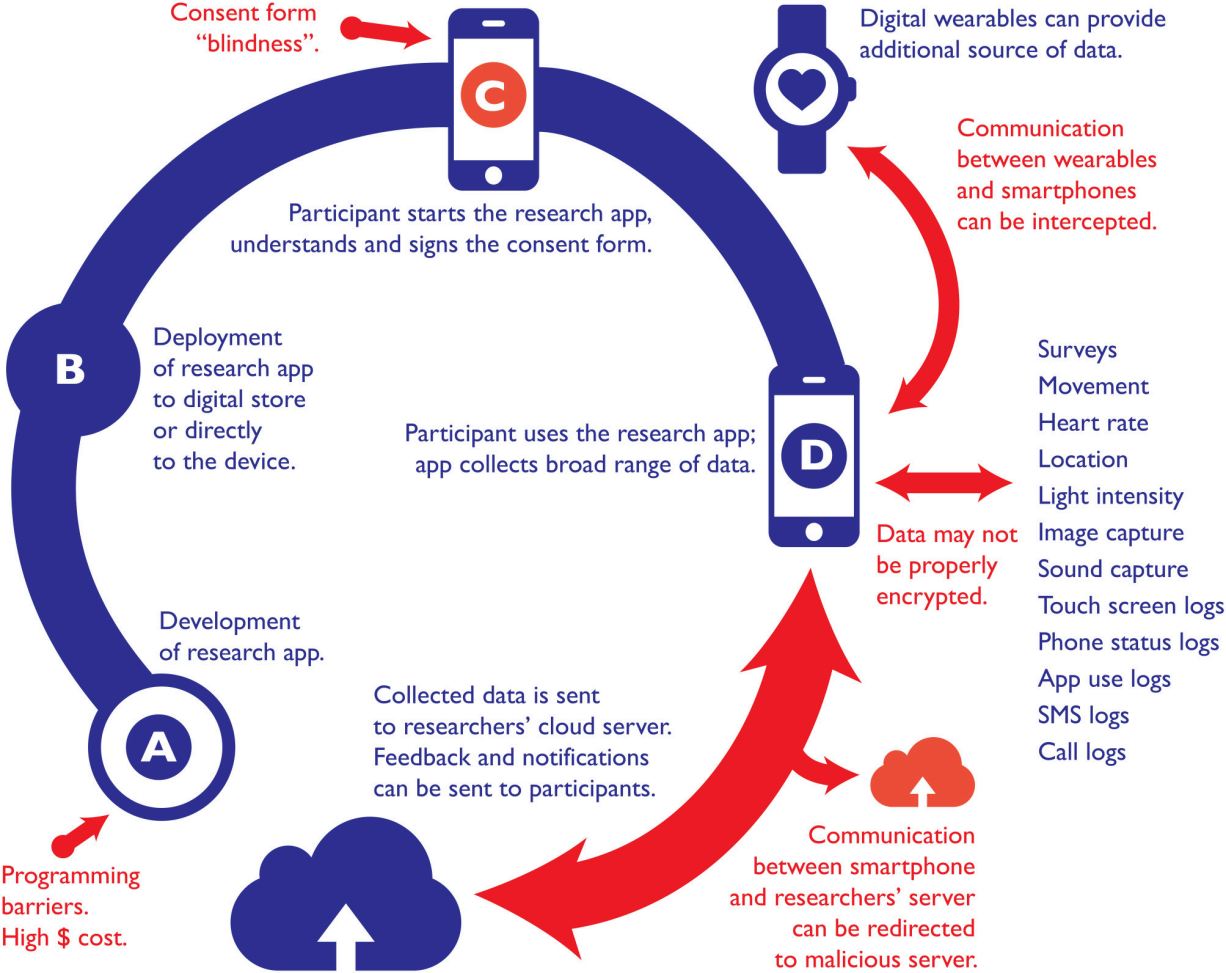
**Diagram showing the life cycle of a smartphone research app: (A) development, (B) deployment to a digital store/device, (C) gaining informed consent, and (D) data collection and transfer**. Practical barriers and problems are highlighted in red.

**Table 1:** **A comparison of frameworks and solutions that have been developed to facilitate the process of creating apps for research purposes**.

	**Type, system, and security**									**Features**						
	**GUI**	**API**	**iOS**	**Android**	**Open source**	**Secured**	**Community**	**Offline**	**Online**	**Notifications**	**Surveys**	**Sensors**	**Wearables**	**Historical**	**Experiments**	**Data vis**
AWARE (Ferreira et al., 2015)	−	+	+	+	+	+	+	+	+	+	+	+	+	−	−	+
Beep me	−	+	−	+	+	−	−	+	−	+	+	−	−	−	−	−
Device analyser (Wagner et al., 2014)	+	−	−	+	+	−	+	−	+	−	−	+	−	−	−	−
EmotionSense (Lathia et al., 2013)	−	+	−	+	+	−	+	−	+	+	+	+	−	−	−	−
Expimetrics	+	−	+	+	−	+	+	−	+	−	+	−^*^	−	−	−	−
Funf (Aharony et al., 2011)	+	+	−	+	+	−	+	+	+	+	+	+	−	−	−	−
Life data	+	−	+	+	−	+	+	−	+	+	+	−^*^	−	−	−	+
MetricWire	+	−	+	+	−	+	+	+	+	+	+	−^*^	−	−	−	−
Momento (Carter et al., 2007)	+	−	+	−	−	+	+	−	+	+	+	−^*^	−	−	−	+
MovisenseXS (Conner and Silvia, 2015)	+	−	−	+	−	+	+	+	+	+	+	−^*^	−	−	−	+
Ohmage (Ramanathan et al., 2012)	+	+	+	+	+	+	+	+	+	−	+	−^*^	−	−	−	+
ResearchKit (Apple, 2016b)	−	+	+	−	+	+	+	−	+	+	+	+	+	+	+	+
SystemSens (Falaki et al., 2011)	−	+	−	+	+	−	+	+	−	−	−	+	−	−	−	−

*Comparison categories (by columns) indicate whether (+) or not (–) a feature is available: (1) GUI—has Graphical User Interface (a type of user interface that allows users to interact with content through graphical icons and visual indicators), (2) API—has Application Programming Interface (a set of functions and procedures that allow the creation of applications which access the features or data of an operating system, application, or other service), (3) iOS—available on Apple iOS system, (4) Android—available on Google Android system, (5) open source—open source (i.e., available to use and modify for free), (6) secured—reasonable level of data security, (7) community—community of users who actively work on addressing issues, debugging, improvement, and support, (8) offline—the study generated with particular solution is available to deploy by direct offline upload to participants' devices, (9) online—the study generated with particular solution is available to deploy via app store or online download, (10) notification—send mobile notification or prompts to study participants, (11) surveys—create surveys, (12) sensors—obtain data from smartphone sensors, (13) wearables—obtain data from sensors beyond the smartphone itself (e.g., via smartwatches), (14) historical—obtain historical data collected within various smartphone systems, (15) experiments—create a active tasks with complex user interactions that utilize systems such as touch screen, camera, microphone and various sensors, (16) data vis—visualize study data or provide summary feedback to participants. Note*:

* *—only GPS sensor is available*.

In theory, solutions with a similar level of flexibility to ResearchKit could provide a robust toolkit for conducing psychological research with smartphones regardless of its methodological complexity. In reality however, new frameworks alone are unlikely to solve the core problems surrounding a lack of psychological engagement with smartphone research. We have identified three barriers that continue to drive the slow adoption of digital smartphone research methods within psychology. These include: (1) programming barriers, (2) consent formblindness', and (3) privacy and security concerns.

## Programming barriers

Programming (writing code) has become a “universal language” for science. Many psychologists already write code in Matlab (Brainard, 1997), Python (Peirce, 2007), and R (Li and Baron, 2012) in order to develop experiments, analyse data, and visualize results. Journals such as Frontiers in Psychology and Behavior Research Methods are a testament to this, with a broad range of code, libraries, and methods freely available and described in detail (e.g., Ellis and Merdian, 2015; Piwek et al., 2015; Sochat et al., 2016). Indeed, ResearchKit was designed to make it much easier to code a research app by providing very specific and accessible tools for users: exhaustive tutorials and manuals, source code with example apps available in open code repository GitHub, and an active support forum (Apple, 2016b). It is possible to create a simple app with those tools and a basic understanding of programming. However, getting the specific details of a ResearchKit app to work in practice remains a daunting task and requires a software developer or computer scientist with the ability to program in Objective C or Swift. Other aspects of app development go far beyond ResearchKit itself as it does not, for example, provide support for data export to a cloud system or storage without additional programming knowledge; at the time of writing, no straightforward solutions exist to get data out of the iOS devices and onto a server using ResearchKit.

If it remains technically difficult to develop apps using available resources, one possibility would be to form interdisciplinary collaborations with computer science departments—who may be more skilled at developing the appropriate software. Otherwise, there are a dearth of programmers available to program a specific app. In the case of psychology, this could result in a researcher outlining their requirements for the app, which a programmer then develops. This method is reasonably unknown, however, and it remains unclear whether this would result in the development of research app that is correctly tuned to methodological and research requirements. Without the ability to see the inner workings of the app, it might be difficult to guarantee that an experimental design works as intended. Finally, any smartphone application might take some time to develop, meaning that development costs run high. According to market research the average cost of developing an app is $270,000 (Formotus, 2016). Cloud services and storage, maintenance and bug fixes also require additional funding and continued development. In the current economic climate, where researchers are increasingly required to demonstrate cost efficient research, this might turn out to an impossible long-term solution.

It is however plausible to assume that programming frameworks such as ResearchKit will gradually become more refined and accessible with more out-of-box' options to deploy research apps without heavy dependence on software developers. Perhaps a solution that will provide a platform for all researchers to develop smartphone apps needs to be more akin to applications that provide a GUI (Graphical User Interface) such as PsychoPy (Peirce, 2007) or SuperLab (Haxby et al., 1993), if the smartphone manifesto is to become a reality. For instance, PsychoPy utilizes a GUI to enable a drag-and-drop' style of interaction where the researcher can directly interact with elements of the study design without programming skills (although PsychoPy still preserves a programming capability with Python to allow for more complex designs). A GUI drastically reduces the complexity of creating, deploying and replicating any study, but has yet to be developed when it comes to building smartphone apps for research purposes.

## Consent form “blindness”

In order for participants to provide informed consent, it is important that they are fully aware of the data that are being collected. There are several problems with ensuring that this happens. Miller (2012) suggests that obtaining ethical approval would be more difficult with smartphone research because people often ignore lengthy “terms and conditions of use” that are necessary when signing up for other online and smartphone services and products. Indeed, a Fairer Finance survey (Daley, 2014) found that of those who did read the terms and conditions, only 17% actually understood the information contained within. While it is unlikely that any information sheet within psychological research would be as complicated and confusing as a 30,000 terms and conditions document, it is worth considering how participants are made aware of and understand what data will be collected about them, how the data will be stored, who will have access to it, and their rights should they wish to withdraw. One solution might involve asking participants (in both smartphone and lab-based research) to read an informed consent form, followed by several related questions. Doing so would ensure that they understand the information and provide authentic informed consent. Long-term developments however, are likely to involve the creation of specific ethical guidelines for the use of smartphones within research. Existing ethical guidelines for internet-mediated research may act as a useful starting point (British Psychological Society, 2013).

## Privacy and security issues

A potentially more problematic issue with regards to ethical practice is the collection, transmission, and storage of data. It is common practice for most smartphone data to be transmitted via WiFi, Bluetooth, cellular network, or NFC, and stored on a cloud server. Data that researchers collect is likely to be sensitive. Indeed, seemingly innocuous data can be used to trace identity (de Montjoye et al., 2013), tell if user is a parent (Seneviratne et al., 2014), detect user mobility patterns (Song et al., 2010), or face-to-face social interactions (Osmani et al., 2014). A recent whitepaper (Symantec, 2014) highlighted that data collected by self-tracking devices and applications can be easily intercepted. While using any smartphone, it remains possible for data to compromised by additional malware applications stored on the phone–although the potential for this has been minimized on Android and iOS devices through the sandboxing of apps. Physical theft of devices can also lead to data being compromised. Transferring data between smartphone and a cloud via WiFi, Bluetooth, cellular network, or NFC may put it at risk of traffic sniffing (allowing attackers access to all transmitted data), and re-direction attacks (which would see data sent to the wrong server). Once stored in the cloud, data are again susceptible to more (and a greater number of) attacks—potentially compromising every user of the specific service (Figure 1D). This therefore makes it difficult to ensure the confidentiality of data.

The outlook may appear bleak when it comes to driving the adoption of smartphones within psychological science however; there are a number of steps that researchers can take to prevent sensitive data becoming compromised. First, data can be encrypted in the device, and only decrypted once the data has been transferred, and is no longer on a cloud device. In the event that data were obtained by unintended recipients, this would render the data practically useless. Also, developing good practice, researchers should minimize the amount of data that is collected. All data collected should relate back to the the specific research questions, and researchers should avoid collecting every retrievable segment of information from a smartphone. Finally, these issues can also be driven back towards the platform provider who should help ensure that data is secured. For example the case here is strong for Apple ResearchKit; Apple highlighted that data protection issue as a critical element for all their products, particularly for sensitive medical data that can be collected with ResearchKit, and they make a noble attempt to protect their devices ecosystem via a range of cryptographic methods and practices (Apple, 2016a). Specifically, differential privacy' aims to maximize the accuracy of queries from users' data while minimizing the chances of identifying an individual by using statistical masking' methods such as hashing, subsampling and noise injection (Dwork and Roth, 2013).

## Conclusion

ResearchKit remains a major topic at Apple's Keynote. All five previous apps were mentioned, and their usefulness demonstrated. While promotional videos suggested that “ResearchKit [had] clearly transformed research” (Apple, 2016a), the reality is rather modest and non- existent outside of medicine. Apple presented only three new apps that were released with the use of ResearchKit in the last year. Thats is not surprising in itself—the event has limited time, but a detailed search reveals that only 25 ResearchKit apps were added between March 2015 and March 2016. In total, only 30 studies have used the ResearchKit system (as for May 2016). Its difficult to evaluate this number because many apps may remain under development, but in comparison there were around 25,000 other apps (Statista, 2016) released in the same period via the App Store. In short, the excitement around ResearchKit for research is high, but adoption remains relatively low across the board.

Smartphone research within psychology remains particularly limited despite clear potential and the practical pains continue to provide significant barriers. First, initial app development is difficult and time intensive even with the advent of standardized development frameworks like ResearchKit. While there are a number of platforms available (and in development) these still require a high level of programming ability. Secondly, ethical issues surrounding data storage and transmission mean that researchers and institutions remain cautious, or unable to provide adequate reassurance that collected data will be secure. A small number of researchers continue to explore the use of smartphones for collecting and validating psychological data, but this has not yet grown into the revolution of psychological and behavioral science research that Miller anticipated in his 2012 manifesto. However, early adopters using smartphone sensors to conduct empirical research have found ways to maintain empirical rigour and demonstrated that many lab-based phenomena are visible when testing outside the lab (Andrews et al., 2015). Therefore, while research within psychology is still not fully benefitting from the power of smartphones, the barriers are perhaps gradually diminishing.

## Author contributions

Conception of the initial idea behind the Perspective: LP, DE. Revisions of consecutive drafts and writing up of final version: LP, DE. Design of Figure 1 diagram: LP.

### Conflict of interest statement

The authors declare that the research was conducted in the absence of any commercial or financial relationships that could be construed as a potential conflict of interest.
