# Corrigendum: Can Programming Frameworks Bring Smartphones into the Mainstream of Psychological Science?

**DOI:** 10.3389/fpsyg.2016.01704

**Published:** 2016-11-02

**Authors:** Lukasz Piwek, David A. Ellis, Sally Andrews

**Affiliations:** ^1^Division of Information, Decisions and Operations, School of Management, University of BathBath, UK; ^2^Department of Psychology, Lancaster UniversityLancaster, UK; ^3^Division of Psychology, Nottingham Trent UniversityNottingham, UK

**Keywords:** smartphones, mobile apps, digital sensors, behavioral informatics, mobile computing

Due to an oversight, Sally Andrews was not included in the author list. The updated author contributions statement appears below. The authors apologize for the mistake. This error does not change the scientific conclusions of the article in any way.

The original article has been updated.

## Author contributions

Conception of the initial idea behind the Perspective: LP, DE. Revisions of consecutive drafts and writing up of final version: LP, DE, SA. Design of Figure 1 diagram: LP.

### Conflict of interest statement

The authors declare that the research was conducted in the absence of any commercial or financial relationships that could be construed as a potential conflict of interest.

